# Unique Reciprocal Association Seen Between Latent Tuberculosis Infection and Diabetes Is Due to Immunoendocrine Modulation (DM-LTB-1)

**DOI:** 10.3389/fmicb.2022.884374

**Published:** 2022-06-27

**Authors:** Vivekanandhan Aravindhan, Anup Bobhate, Kuppan Sathishkumar, Aruna Patil, Satyavani Kumpatla, Vijay Viswanathan

**Affiliations:** ^1^Department of Genetics, Dr. ALM PG IBMS, University of Madras, Chennai, India; ^2^Prof. M. Viswanathan Diabetes Research Centre, Chennai, India; ^3^ESIC-PGIMSR Medical College and Hospital, Chennai, India

**Keywords:** diabetes, hypertension, inflammation, quantiferon, cytokines, latent tuberculosis infection

## Abstract

**Aim:**

The prevalence of latent tuberculosis infection (LTBI) among diabetes patients is poorly studied. In the present study, the prevalence of LTBI among pre-diabetes and diabetes patients was studied, along with immunoendocrine biomarkers (*n* = 804).

**Methods:**

LTBI was screened by Quantiferon TB gold in Normal glucose tolerance [(NGT); *n* = 170, [Pre-diabetes (PDM; *n* = 209), Newly diagnosed diabetes (NDM; *n* = 165) and Known diabetes (KDM; *n* = 260) subjects. CRP, TNF-α, IL-6, IL-1β, IFN-β, IL-12, IFN-γ, IL-2, insulin, leptin, and adiponectin levels in serum and IFN-γ levels in quantiferon supernatants were quantified by ELISA. The expression of T-bet was quantified using qRT-PCR. Serum TBARS and nitrite levels were quantified by colorimetry.

**Results:**

The LTBI prevalence was 32% in NGT, 23% in PDM, 24% in NDM, and 32% in KDM groups, with an adjusted OR of 0.61 (*p* < 0.05). Downregulation of CRP, TNF-α, and nitrites and upregulation of adiponectin could be responsible for LTBI mediated protection against insulin resistance (IR), while the high levels of IL-1β, IL-12, and leptin could be responsible for IR mediated anti-TB immunity. The defective antigen-specific IFN-γ response, as seen in the KDM group, could be responsible for the low detection rate of LTBI and high probability of endogenous reactivation.

**Conclusion:**

There appears to be a biphasic relationship between diabetes-latent tuberculosis: At the early stages of diabetes it is reciprocal, while at a late stage it is synergistic, this important phenomenon obviously needs further research.

## Introduction

Global epidemiological studies have clearly shown increased susceptibility of diabetes patients to tuberculosis (TB), which is often called “Diabetes-Tuberculosis Synergy” (Jeon and Murray, 2008; Sen et al., 2009). TB patients with diabetes, present a higher bacillary load in the sputum, delayed sputum conversion, and higher rates of multidrug-resistant (MDR-TB) infection (Jeon and Murray, 2008; Sen et al., 2009). They also present with higher lung cavitary involvement, compared to their non-diabetes counterparts (Jeon and Murray, 2008; Sen et al., 2009). These results imply that patients with TB and diabetes, may be more seriously ill, and may pose a higher risk for spread, in the community (Jeon and Murray, 2008; Sen et al., 2009). Recent epidemiological surveys have clearly shown the possibility of diabetes–tuberculosis (DM-TB) nexus in near future, which needs immediate attention (Jeon and Murray, 2008; Sen et al., 2009). Chronic inflammation has long been identified as a risk factor for diabetes (Martinez and Kornfeld, 2014; Ayelign et al., 2019). Inflammation in diabetes is low-grade, systemic, and non-antigen-specific in nature (Martinez and Kornfeld, 2014; Ayelign et al., 2019). Most importantly, it impairs immunity and makes them more susceptible to TB (Martinez and Kornfeld, 2014; Ayelign et al., 2019). Diabetes subjects also have increased redox stress which fuels inflammation and impairs immunity (Yuan et al., 2019). Diabetes in Asian Indians is characterized by the younger age of onset, lower body mass index, propensity for central obesity, increased fat/muscle ratio, and increased insulin resistance (compared to other ethnic populations), which has been described as the “Asian–Indian Phenotype” (Mohan and Deepa, 2006). Whether the Asian-Indian phenotype (which is not fully characterized) is also a risk factor for TB is not known.

A substantial increase in diabetes is taking place in the world, where approximately one-third of the population is latently infected with *Mycobacterium tuberculosis* (latent tuberculosis infection-LTBI) (Lee et al., 2016). The increased incidence of TB in patients with diabetes, could either be due to the reactivation of latent tuberculosis (endogenous reactivation) or due to new infection (exogenous re-infection), which is the consequence of the weakening of the immune system (Lee et al., 2016). The Diabetes–Tuberculosis synergy is due to a vicious cycle: The tubercle bacillus induces a strong inflammatory response and redox stress which can worsen insulin resistance (Nishio et al., 1988) and pancreatic beta-cell loss (Park et al., 2014), precipitating diabetes among pre-diabetics, or worsening glucose intolerance in chronic diabetics (Mahat et al., 2019). On the other hand, chronic diabetes weakens the immune system, increasing the chances of TB infection among uninfected (exogenous infection), or augmenting re-activation, in those who are latently infected (endogenous reactivation) (Nematollahi et al., 2020). Compared to active TB in diabetes, LTBI in diabetes is poorly studied (Lee et al., 2016). Further with respect to diabetes, most studies have been carried out only on chronic diabetes subjects and what happens during the pre- and early stages of diabetes is still an enigma. Recently, we reported stage-specific modulation of inflammation, by LTBI, among subjects with various grades of glucose intolerance (Bobhate et al., 2021; Aravindhan et al., 2022). As a logical next step, in the present study, we looked at the prevalence of LTBI, at different stages of glucose intolerance. Diabetes is often associated with obesity, hypertension, and dyslipidemia (Lee et al., 2016). Thus, we took a holistic approach to study the association between metabolic co-morbidities, often seen with diabetes, with LTBI. The immunoendocrine mechanism behind DM–LTB synergy was studied by systemic cytokine and hormone profiling. Systemic redox stress was studied by quantifying circulating levels of lipid peroxidation products (oxidative stress) and nitrites (nitrosative stress). Finally, anti-TB immunity was studied using antigen specific IFN-γ response and T-bet expression in LTB^+^ subjects.

## Materials and Methods

### Study Protocol

**Figure 1:**
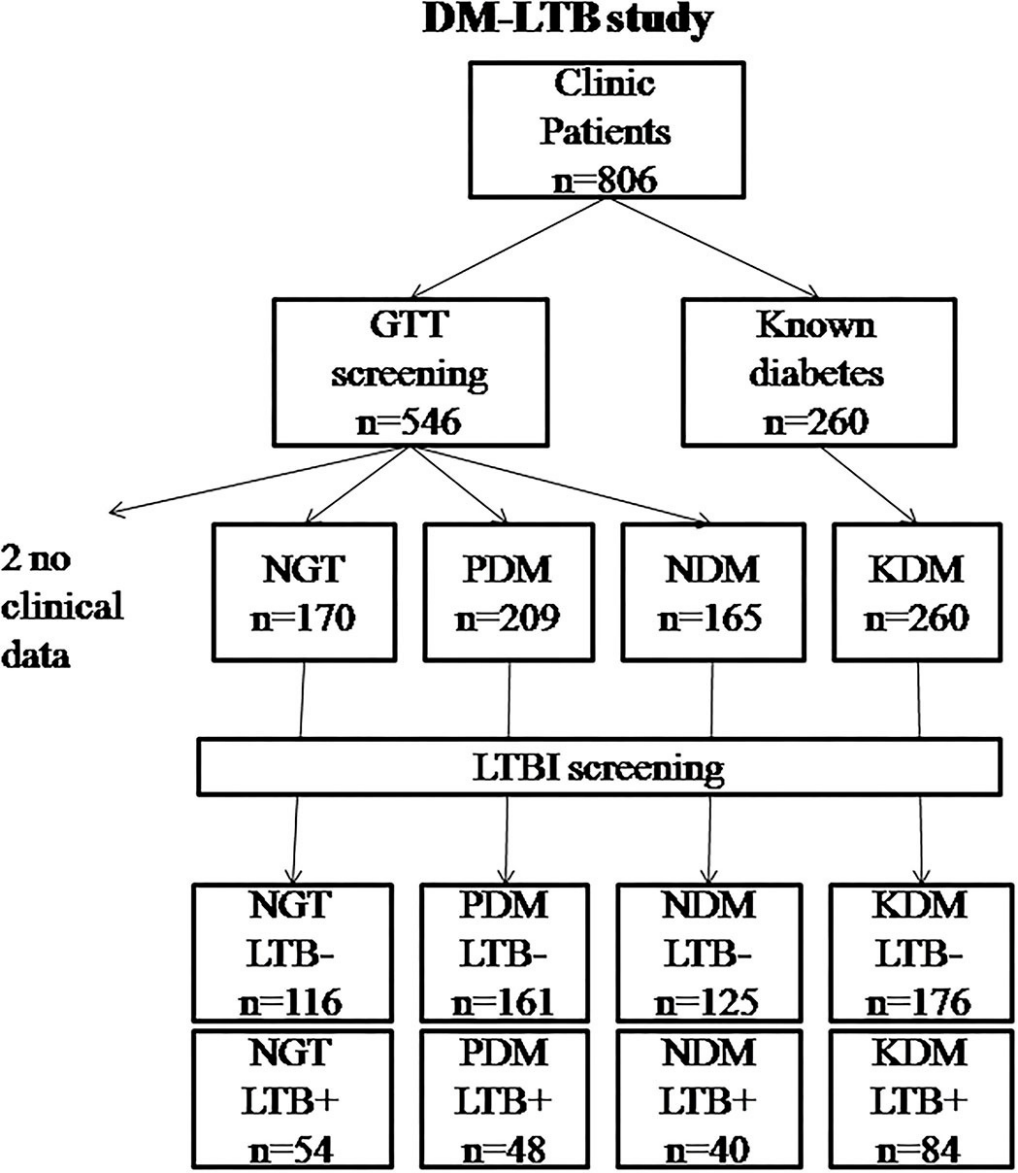
Flowchart showing the recruitment of study subjects into the DM-LTB study. LTB screening was carried out using IGRA assay. Diabetic status was determined based on oral glucose tolerance test as per WHO criteria. For two patients, clinical data couldn't be obtained and hence were removed from analysis.

A total of 804 subjects were recruited for this study (from 2016 to 2019) (Figure 1). The study subjects were selected from the outpatient department of M.V. Hospital for Diabetes, a tertiary care center for diabetes in South India. The study protocol was approved by the institutional ethics committee (Ref No-MVH/IHEC/02/2013/006). Written informed consent was obtained from all the study participants. The study was conducted as per the Declaration of Helsinki and STROBE guidelines.

#### Study Design

Case controlled, cross-sectional, observational study.

#### Screening for Diabetes

The study subjects were divided into four groups based on their glycemic profile (fasting blood glucose, 2-Hr blood glucose, postprandial blood glucose, and HbA1c levels), as per WHO criteria, as reported previously (Viswanathan et al., 2012; Kornfeld et al., 2020).

#### Definition

1. Normal glucose tolerance (NGT; *n* = 170): FPG < 100 mg/dL (5.6 mmol/L) and 2-Hr PG < 140 mg/dl (7.8 mmol/L)
2. Pre-diabetes (PDM; *n* = 209): FPG-100–125 mg/dl (5.6–7.0 mmol/L -) and/or 2-Hr PG- 140–199 mg/dl (7.8–11.1 mmol/L)
3. Newly diagnosed diabetes (NDM; *n* = 165): ≥126 mg/dL (7.0 mmol/L) and/or 2-Hr PG ≥200 mg/dL (11.1 mmol/L)
4. Known diabetes (KDM; *n* = 260): Patients who self-reported diabetes and are under treatment.

### Screening of LTBI

All the study subjects were screened for LTBI using the QuantiFERON-TB Gold test, as per the kit protocol. Briefly, 1 ml of whole blood was collected in QFT tubes per-coated with saline (nil control) or TB antigens (ESAT-6, CFP-10, and TB7), and incubated for 16 h at 37°C. After the incubation period, the supernatant was separated and QFT-GIT-ELISA was carried out, as per the manufacturer's instructions. The test results were interpreted as per kit guidelines, using the software provided by the manufacturer. The subjects were considered LTB^+^ if the QFT results were positive.

### Inclusion and Exclusion Criteria

The inclusion criteria were adult subjects with normal blood count and hemoglobin levels. The exclusion criteria were patients with type-1 diabetes and those previously diagnosed with TB, cancer patients, pregnant women, and those on steroids or antibiotics.

### Anthropometric and Biochemical Parameters

Anthropometric measurements including height, weight, and waist circumference were obtained using standardized techniques (Viswanathan et al., 2012; Kornfeld et al., 2020). Glycemic parameters (fasting blood glucose and 2 h glucose), lipid parameters (LDL, HDL, VLDL, TGL, and total cholesterol), and renal parameters (urea and creatinine) were measured using a fully automated autoanalyzer BS400 (Mindray, China). Glycated hemoglobin (HbA1c) was measured by HPLC (Bio-Rad, Hercules, CA, USA). The intra- and inter-assay coefficient of variation for the biochemical assays ranged between 3.1 and 5.6%.

### Quantification of Serum Cytokines and Hormones

Cytokine and hormone measurements were carried out in the stored serum and quantiferon supernatants (both stored at −80°C), in a subgroup of the study subjects. All the LTB^+^ PDM and NDM subjects and equal number of age and gender matched, LTB^−^ subjects were included for serum cytokine analysis: PDM = 70 (PDM-LTB^−^ = 37, PDM-LTB^+^ = 33) and NDM = 56 (NDM-LTB^−^ = 31, NDM-LTB^+^ = 25). IFN-γ levels in the Quantiferon tubes of NGT-LTB^+^ (*n* = 50), PDM-LTB^+^ (*n* = 47), NDM-LTB^+^ (*n* = 39) and KDM-LTB^+^ (*n* = 76) subjects were estimated by ELISA. The serum levels of CRP, pro-inflammatory cytokines (TNF-α, IL-6, and IL-1β), adaptive immune cytokines (IL-12, IFN-γ, and IL-2), type-1 interferon (IFN-β) and hormones (insulin, leptin and adiponectin) were estimated by ELISA, as per the kit protocol (R&D System, USA). The lowest detection limits for the tested cytokines were: CRP- 781.25, TNF-α-1.95, IL-6-0.59, IL-1β-0.24, IL-12p70-1.95, IFN-γ-1.17, IL-2-1.95, and IFN-β-7.81, Insulin- 15.5 pmol/L, Adiponectin- 0.89 μg/ml, and Leptin- 5.8 ng/ml.

### Estimation of Plasma Nitrate Levels and Redox Stress

The level of nitrate in serum was estimated using Griess reagent (SRL Chem, India) and absorbance was measured at 540 nm. Levels of TBARS (redox stress markers) were measured in the serum using a commercially available kit (Cayman Chemicals, USA), following manufacturer's protocol.

### Peripheral Blood Leukocyte Cultures

Whole blood cultures from the study subjects were performed as described previously (Al-Nimer et al., 2012). Briefly, whole blood was collected in EDTA coated tubes. After centrifugation, the packed cell volume was diluted with RPMI medium (1:1 ratio) containing 10% FCS and was used for *in vitro* culture. Cells were stimulated with Purified Protein Derivative (10 μg/ml) (PPD) and *Mycobacterium tuberculosis H37Rv* whole cell lysate (10 μg/ml) (WCL) or were left unstimulated (control), for 72 h. The cell pellets were solubilized in RNAzol and were stored at −80°C. WCL and PPD were kindly gifted by Dr. D. Anbarasu and Dr Luke Elizabeth Hanna from NIRT(ICMR), Chennai, respectively.

### Real-Time PCR Analysis

RNA extraction from the stored samples was carried out using RNeasy Mini Kit (Qiagen). The quality and quantity of the extracted RNA was quantified using nanodrop. About 1 μg of RNA was reverse transcribed to cDNA and real-time PCR was performed using TaqMan probes (Applied Biosystems, US) specific for T-Bet/TBX21 (Assay ID: Hs00894392_m1). 18S rRNA was used as a housekeeping control. Gene expression levels (normalized to 18S rRNA) were analyzed using the StepOnePlus RT-PCR system (Applied Biosystems, Foster City, CA, USA) and 2^−ΔΔCt^ was calculated for all samples with unstimulated sample values as reference.

### Statistical Analyses

Student's *t*-test was used to compare groups for continuous variables that followed a normal distribution, whereas one-way ANOVA was used to compare the subgroups. The Kruskal–Wallis test was used for comparing cytokine levels. The Pearson's *X*^2^-test or Yate's correction test (as appropriate) was used to compare proportions. Logistic regression analysis was used to quantify the association between metabolic morbidities and LTBI. Pearson's correlation analysis was carried out to find the association between clinical parameters and serum biomarkers. Multiple comparisons were corrected using Holm's correction for each set of analyses. All the analyses were conducted using SPSS software and *p* < 0.05 was considered significant.

## Results

### Clinical Characteristics of the Study Subjects

Table 1 shows the clinical and biochemical characteristics of the study groups. No significant difference was seen in age, gender, and BMI between the groups. Interestingly, in all the four groups (NGT, PDM, NDM, and KDM) the SBP and DBP values were higher in LTB^+^ compared to LTB^−^ sub-groups and reached statistical significance under certain conditions. The FPG levels were significantly higher in both NDM and KDM groups compared to NGT and PDM groups. The glycemic parameters were significantly higher in PDM, NDM, and KDM compared to the NGT group. No significant difference was seen in glycemic parameters between LTB^−^ and LTB^+^ subgroups. TGL levels were higher in NDM and KDM groups, and HDL levels were low in the KDM group. VLDL levels were higher in LTB^+^ compared to LTB^−^ NGT group. No significant change was seen in urea and creatine levels between groups. The disease duration, drug regimen, and complications were not significantly different between LTB^+^ and LTB^−^ KDM subgroups.

**Table 1:** Clinical characteristic of the study groups.

**Groups**	**NGT (*n =* 170)**		**PDM (*n =* 209)**		**NDM (*n =* 165)**		**KDM (*n =* 260)**	
**Clinical parameters**	**NGT-LTB^−^(*n =* 116)**	**NGT-LTB^+^ (*n =* 50)**	**PDM-LTB^−^(*n =* 161)**	**PDM-LTB^+^ (*n =* 47)**	**NDM-LTB^−^(*n =* 125)**	**NDM-LTB^+^ (*n =* 39)**	**KDM-LTB^−^(*n =* 176)**	**KDM-LTB^+^ (*n =* 76)**
Age (years)	45.3 ± 11.3	50. ± 10.9	49.1 ± 10.2	49.9 ± 9.3	48.80 ± 11.3	45.90 ± 10.7	52.88 ± 9.5	53.14 ± 9.9
Gender (M/F)	59/57	33/21	91/70	25/23	82/43	30/10	120/56	65/19
BMI(kg/m^2^)	27.6 ± 4.5	26.9 ± 4.2	29.7 ± 8.1	28.7 ± 3.6	28.4 ± 4.4	27.9 ± 4.3	27.3 ± 4.6	28.6 ± 5.7
SBP (mm Hg)	120.4 ± 16.1	126.2 ±17.9^*a^	122.9 ± 13.2	128.4 ±16.3* ^a^	126 ±15.8^*^ ^b^	129.9 ± 18.8	128.1 ±16.4^***^ ^b^	130.5 ± 16.4
DBP (mm Hg)	77.5 ± 8.9	82.4 ±9.8^**^ ^a^	78.2 ± 8.9	79.7 ± 10.6	79.3 ± 9.1	82.4 ±9.8^*^ ^a^	78.5 ± 8.4	80 ± 8.1
FPG (mg/dL)	91 ± 7.5	90 ± 5.9	105.3 ± 15.7	104.6 ± 13.7	169.1 ±60.8^***^ ^b^	175.7 ±58.3^***^ ^c^	181.9 ±76.9^***^ ^b^	158.7 ±57.7^*^^a^, ***^c^
2Hr-PG/PP (mg/dL)	112 ± 21.6	110.7 ± 22.5	162 ±35.7^***^ ^b^	163.1 ±29.4^**^ ^c^	315.9 ±107.5^***^ ^b^	334.6 ±95.4^***^ ^c^	280.9 ±102.7^***^ ^b^	276.7 ±93.90^***^ ^c^
HbA1c level (%)	5.4 ± 0.7	5.4 ± 0.3	5.9 ±0.5^***^ ^b^	5.9 ±0.4^***^ ^c^	8.2 ±1.9^***^ ^b^	8.6 ±1.9^***^ ^c^	8.9 ±2.0^***^ ^b^	9 ±1.9^***^ ^c^
Cholesterol (mg/dL)	186.1 ± 36.7	190.6 ± 30.2	185.5 ± 38.6	192.6 ± 46.4	199.7 ± 44.8	199.2 ± 39	171.3 ±43.7^*^ ^b^	173.5 ±46^*^ ^c^
TGL (mg/dL)	111 ± 47.7	106.9 ± 44.6	131.3 ± 63.3	140.7 ± 82.7	166.9 ±101.7^***^ ^c^	166.3 ±128.4^***^ ^d^	142.3 ±100.3^*^ ^c^	166 ±225.1^*^ ^d^
HDL (mg/dL)	44.2 ± 9.5	46.4 ± 8.2	42.7 ± 8.8	43.1 ± 8.2	42.8 ± 9.1	42.2 ± 8.5	41.3 ±15*^c^	39.8 ±9.5^***^ ^d^
LDL (mg/dL)	111.8 ± 25.6	114.2 ± 23.2	111 ± 28.7	115 ± 29.5	119.6 ± 30.5	121 ± 22.8	100.6 ±28.4^*^ ^c^	101.4 ± 29.4
VLDL (mg/dL)	30.6 ± 13.5	36.8 ±18.9^*^ ^a^	30.9 ± 10.6	34.4 ± 16.8	36.6 ±14.2^**^ ^c^	36.8 ± 18.9	30.5 ± 12.6	32.7 ± 16.4
Urea (mg/dL)	22.2 ± 6.6	22.9 ± 8.2	23.2 ± 7.5	22.7 ± 7.7	23.1 ± 9.2	26 ± 23.3	24.7 ± 9.9	24.3 ± 10.5
Creatnine (mg/dL)	0.9 ± 0.1	1 ± 0.1	1 ± 0.2	1 ± 0.5	1 ± 0.1	1 ± 0.1	1 ± 0.6	1.1 ± 0.3
Disease duration	NA	NA	NA	NA	NA	NA	9.7 ± 7.1	9.4 ± 6.3
Drug regimen	NA	NA	NA	NA	NA	NA	Insulin (29%), Metformin (60%), sulfonylurea (50%) and DPP4 inhibiotrs (15%) and other (38%)	Insulin (19%), Metformin (70%), sulfonylurea (58%) and DPP4 inhibiotrs (20%) and other (41%)
Complications	NA	NA	NA	NA	NA	NA	Nephropathy (13%), Neuropathy (7%); Retinopathy (14%); Dyslipidaemia (27%) Hypertension (11%)	Nephropathy- (19%), Neuropathy- (2%); Retinopathy- (18%); Dyslipidaemia- (27%) Hypertension- (17%)

*In the data mentioned above continuous variables are mentioned as mean ± SD and proportions as percentage*.

*Numbers highlighted in bold are significant values*.

*BMI, Body Mass Index; SBP, Systolic Blood Pressure; DBP, Diastolic Blood Pressure; FPG, Fasting Plasma Glucose; 2Hr, PG/PP- 2Hrs Post glucose or Post Prandial Glucose; HbA1c, Glycated Hemoglobin; HDL, High Density Lipoprotein; LDL, Low Density Lipoprotein; VLDL, Very Low Density Lipoprotein; TC, Total Cholesterol; TGL, Triglyceride lipids; N/A, Not applicable*.

*Comparison between LTB^−^ vs. LTB^+^ groups using Student t-test*.

*LTB^−^ subjects of PDM, NDM and KDM groups were statistically compared with NGT using One-way ANOVA test*.

*LTB^+^ subjects of PDM, NDM and KDM groups were statistically compared with NGT using One-way ANOVA test*.

### Association of Clinical Parameters with LTBI

**Figure 2:**
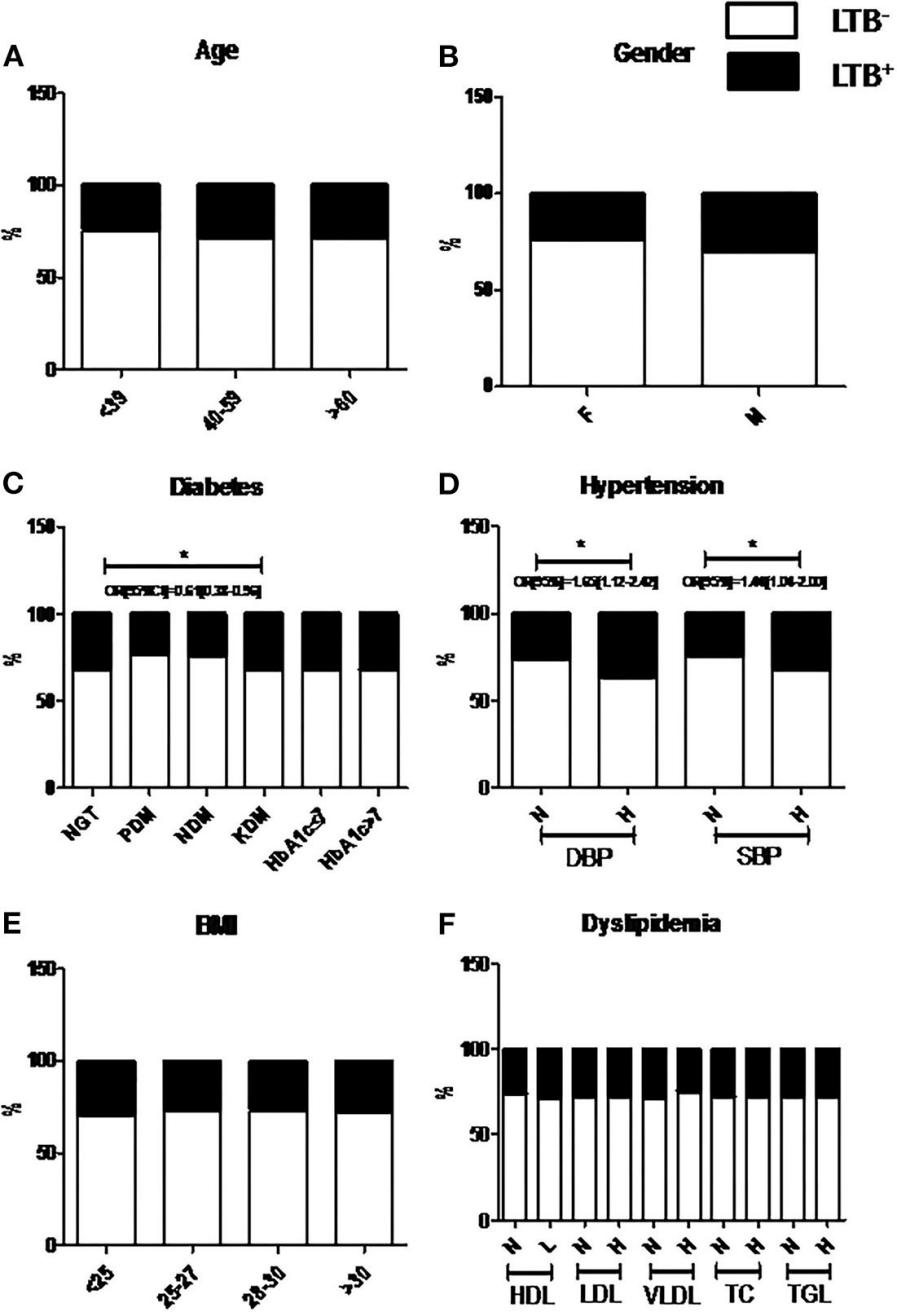
Prevalence of LTBI was significantly decreased among NDM and PDM groups compared to NGT and KDM groups, while the prevalence is significantly increased among the hypertensive groups. The study subjects were stratified based on Age **(A)**, Gender **(B)**, Diabetes **(C)**, Hypertension **(D)**, BMI **(E)** and Dyslipidemia **(F)** and the percentage of LTB^−^ and LTB^+^ subgroups, within each group were plotted. *P* < 0.05 was considered significant. *P*-value and OR[95%CI] were calculated using chi-square analysis and binary regression analysis, respectively. LTB, Latent Tuberculosis; BMI, Body Mass Index; SBP, Systolic Blood Pressure; DBP, Diastolic Blood Pressure; FPS, Fasting Plasma Glucose; PPPG, Post Prandial Plasma Glucose; HbA1c, Glycated Hemoglobin; HDL, High Density Lipoprotein; LDL, Low Density Lipoprotein; VLDL, Very Low Density Lipoprotein; TC, Total Cholesterol; TGL, Triglyceride lipids; NS, Non-significant.

Figure 2 shows the Chi-square analysis between metabolic morbidities and LTBI. The stratification of subjects based on anthropometric and biochemical cut-off values was done as per WHO criteria, which were followed previously (Viswanathan et al., 2012; Kornfeld et al., 2020). No significant association was seen between age, gender, or BMI with LTBI (Figures 2A,B,E). Interestingly, the percentage of LTBI was found to be significantly lower in PDM (23%) and NDM (24%) compared to NGT (32%) and KDM (32%) groups, with an adjusted OR of 0.61 [0.38–0.96] (*p* < 0.05) (Figure 2C). However, no significant difference was seen with respect to the LTBI percentage between the NGT and KDM groups (Figure 2C). Another interesting finding is the strong association seen between hypertension and LTBI (Figure 2D). The percentage of LTBI was significantly higher in the hypertensive compared to non-hypertensive groups (non-hypertensive- 37% vs. diastolic hypertensive-29%; non-hypertensive- 32% vs. systolic hypertensive-28%) (OR: DBP-1.7 [1.12–2.4] and SBP-1.4 [1.04–2.0]; *p* < 0.05) groups (Figure 2D). No significant association was seen between the lipid parameters and LTBI (Figure 2F).

### Decreased Prevalence of LTB in PDM was Associated with Decreased CRP and TNF-α and Increased Adiponectin Levels

**Figure 3:**
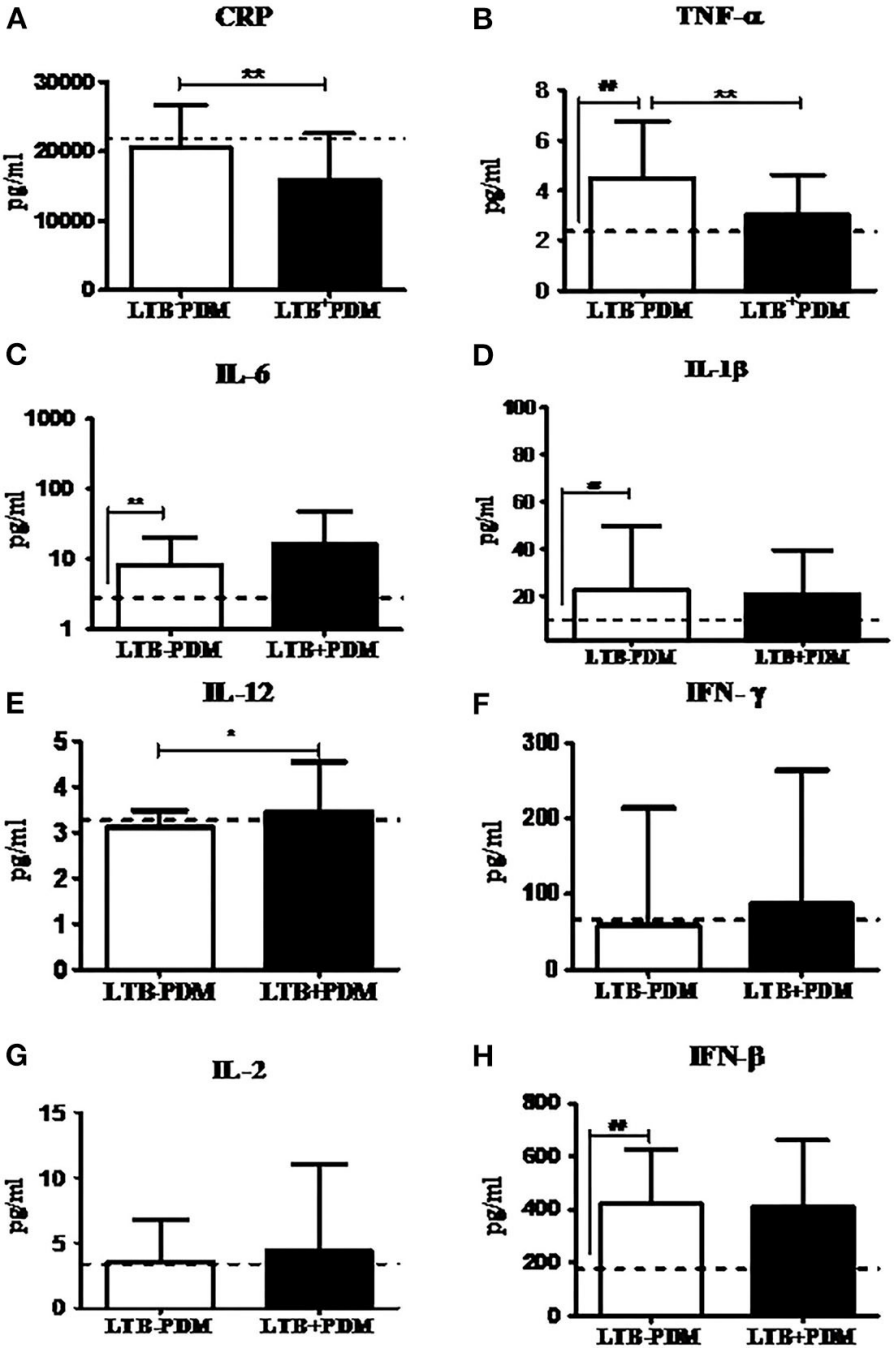
LTBI reduces CRP and TNF-α and increases IL-12 levels in PDM subjects. The circulating levels of pro- inflammatory cytokines, Th1 cytokines and type-1 interferon in PDM subjects were quantified by ELISA. Bar graph showing the circulating levels of CRP **(A)**, TNF-α **(B)**, IL-6 **(C)**, IL-1β **(D)**, IL-12 **(E)**, IFN-γ **(F)**, IL-2 **(G)**, and IFN-β **(H)** in the serum of LTB+PDM (*n* = 47) and LTB-PDM (*n* = 47) subjects. The basal level, as was seen in LTB-NGT, is shown as dashed line (—-), as reference. Statistical significance was determined by a non-parametric Mann–Whitney *U*-test and *p* < 0.05 was considered significant. *p < 0.05; **p < 0.01 for LTB-PDM vs. LTB+PDM; **##** *p* < 0.01 for LTG-NGT vs. LTB-PDM.

**Figure 4:**
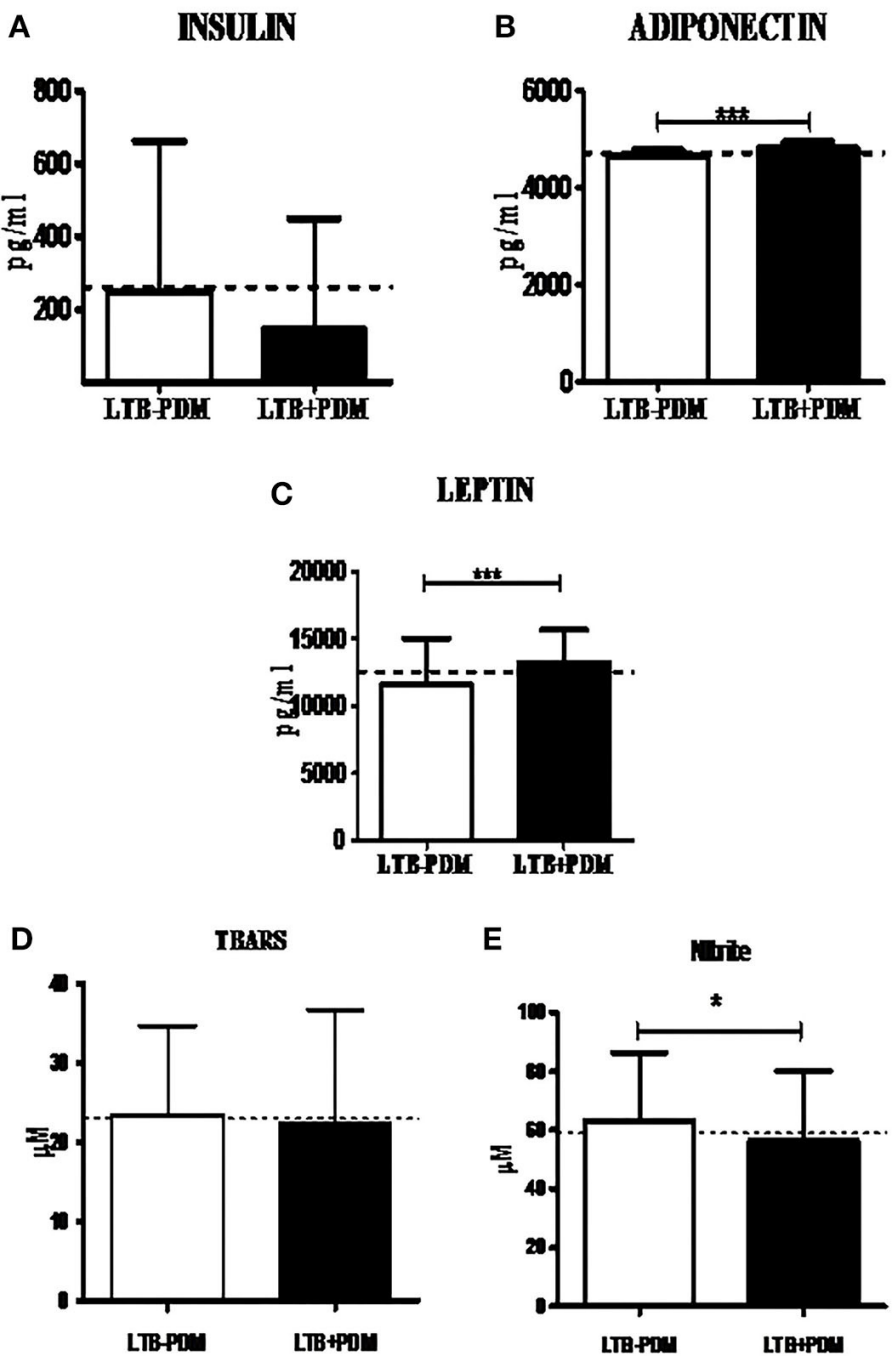
LTBI increases Leptin and Adiponectin levels and decreases nitrite levels in PDM subjects. The circulating levels of hormones and redox markers in PDM subjects were quantified by ELISA and colorimetry, respectively. Bar graph showing the circulating levels of Insulin **(A)**, Adiponectin **(B)** and Leptin **(C)**, TBARS **(D)**, and nitrite **(E)** levels in the serum of LTB+PDM (*n* = 47) and LTB-PDM (*n* = 47) subjects. The basal level, as was seen in LTB-NGT, is shown as dashed line (—-), as reference. Statistical significance was determined by non-parametric Mann–Whitney *U*-test and *p* < 0.05 was considered significant. *p < 0.05; ***p < 0.001 for LTB-PDM vs. LTB+PDM.

Next, in order to study the decreased prevalence of LTB in the PDM group, systemic cytokine (Figure 3), hormone, and redox marker (Figure 4) profiling was carried out, on LTB^−^ and LTB^+^ PDM subjects. Figure 4A shows the serum levels of CRP, innate immune (TNF-α, IL-6, IL-1β, and IFN-β), and adaptive immune cytokines (IL-12, IFN-γ, and IL-2), in LTB^−^ and LTB^+^ PDM subjects. CRP (Figure 3A) and TNF-α (Figure 3B) levels were significantly reduced, in LTB^+^PDM compared to LTB^−^PDM subjects. With respect to hormone levels, serum leptin (Figure 4B) and adiponectin (Figure 4C) levels were significantly increased in LTB^+^PDM compared to LTB^−^PDM subjects. While no significant difference was seen in the TBARS levels (Figure 4D), nitrite levels were significantly reduced in LTB^+^PDM, compared to LTB^−^PDM subjects (Figure 4E).

### Decreased Prevalence of LTB in NDM was Associated with Increased IL-1β and Adiponectin Levels

**Figure 5:**
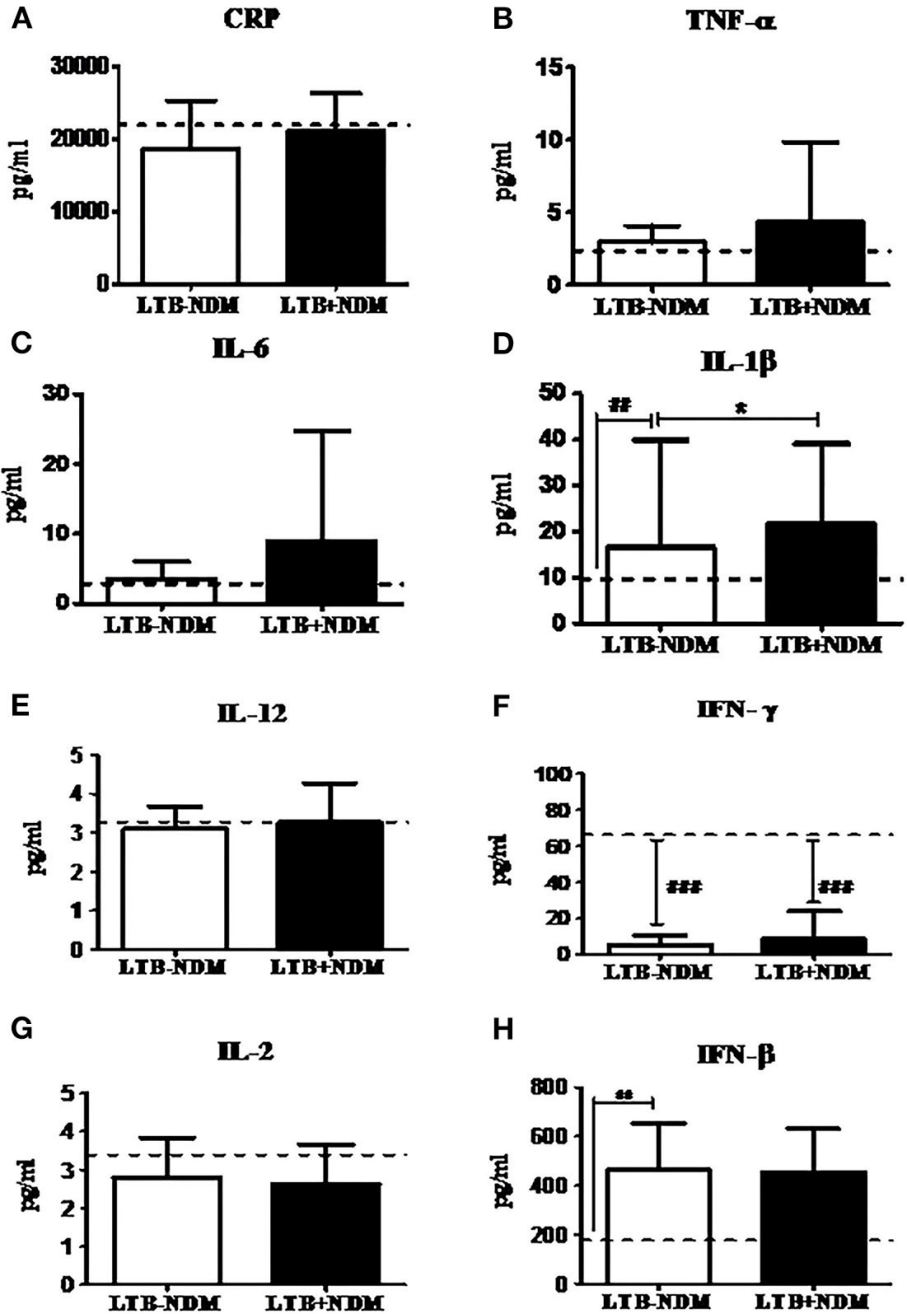
LTBI increases IL-1β levels in NDM subjects. The circulating levels of pro- inflammatory cytokines, Th1 cytokines, and type-1 interferon in NDM subjects were quantified by ELISA. Bar graph showing the circulating levels of CRP **(A)**, TNF-α **(B)**, IL-6 **(C)**, IL-1β **(D)**, IL-12 **(E)**, IFN-γ **(F)**, IL-2 **(G)**, and IFN-β **(H)** in the serum of LTB-NDM (*n* = 39) and LTB+NDM (*n* = 39) subjects. The basal level, as was seen in LTB-NGT, is shown as dashed line (—-), as reference. Statistical significance was determined by non-parametric Mann–Whitney U test and *p* < 0.05 was considered significant. *p < 0.05; **p < 0.01 for LTB-PDM vs. LTB+PDM; **##** *p* < 0.01 for LTG-NGT vs. LTB-PDM.

**Figure 6:**
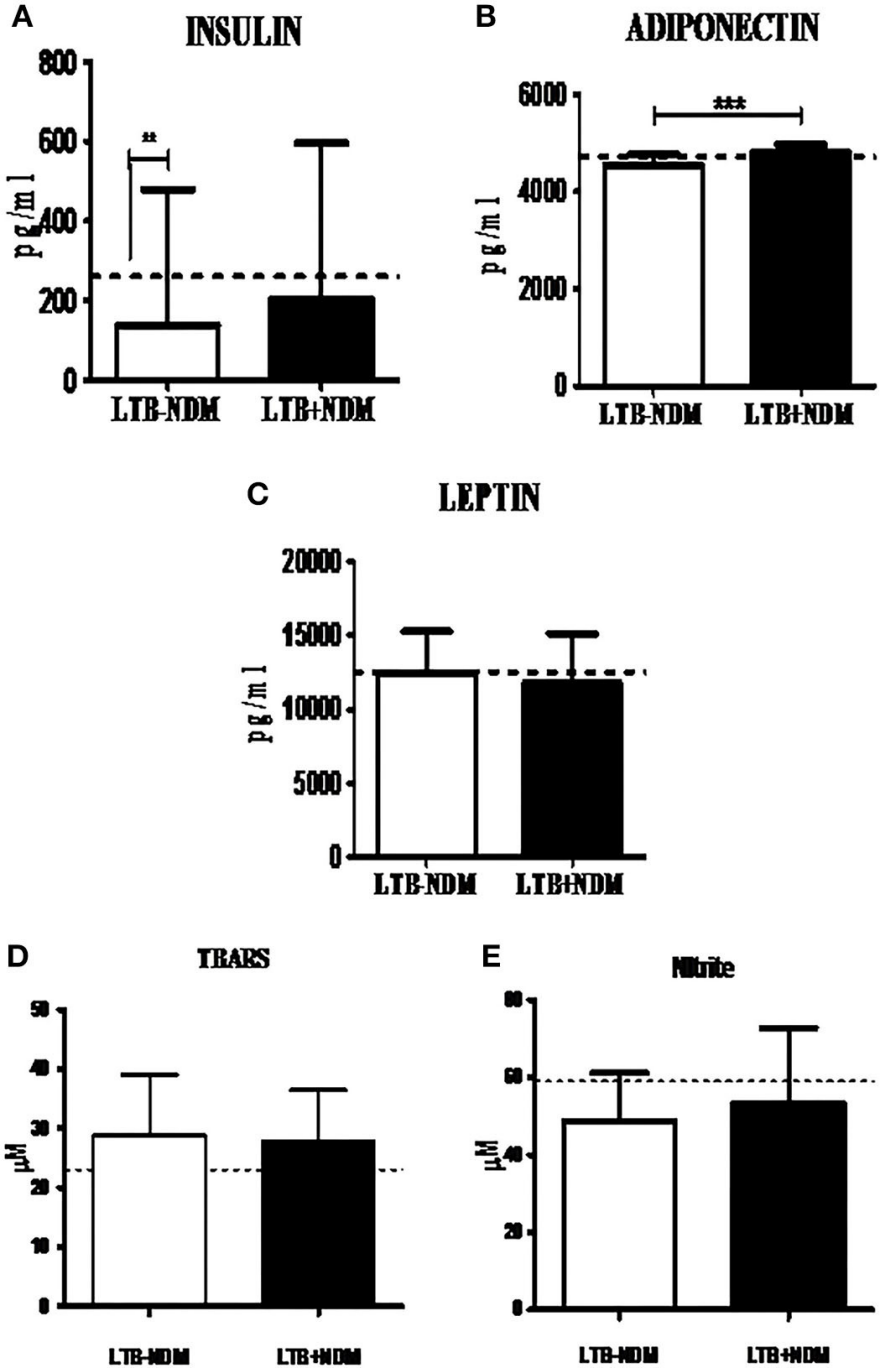
LTBI increases Adiponectin levels in NDM subjects. The circulating levels of hormones and redox markers in PDM subjects were quantified by ELISA and colorimetry, respectively. Bar graph showing the circulating levels of Insulin **(A)**, Adiponectin **(B)**, Leptin **(C)**, TBARS **(D)**, and nitrite **(E)** levels in the serum of LTB-NDM (*n* = 39) and LTB+NDM (*n* = 39) subjects. The serum levels of TBARS and nitrites were quantified using colorimetry. The basal level, as was seen in LTB-NGT, is shown as dashed line (—-), as reference. Statistical significance was determined by non-parametric Mann–Whitney *U*-test and *p* < 0.05 was considered significant. *p < 0.05; **p < 0.01 for LTB-NDM vs. LTB+NDM; **##** *p* < 0.01 for LTB-NGT vs. LTB-NDM.

Next, in order to study the decreased prevalence of LTB in the NDM group, systemic cytokine (Figure 5) and hormone (Figure 6) profiling was carried out, on LTB^−^ and LTB^+^ NDM subjects. Figure 5 shows the serum levels of CRP, innate immune (TNF-α, IL-6, IL-1β, and IFN-β), and adaptive immune cytokines (IL-12, IFN-γ, and IL-2), in LTB^−^ and LTB^+^ NDM subjects. Only IL-1β levels (Figure 5A) were significantly increased in LTB^+^NDM, compared to LTB^−^NDM subjects. With respect to hormone levels, serum adiponectin (Figure 6C) levels were significantly increased in LTB^+^NDM compared to LTB^−^NDM subjects. No significant difference was seen in the TBARS (Figure 6D) and nitrite (Figure 6E) levels between LTB^+^NDM and LTB^−^NDM subjects.

### Lack of Increased Prevalence of LTBI in KDM was Associated with Decreased Secretion of IFN-γ in the Culture Supernatant

**Figure 7:**
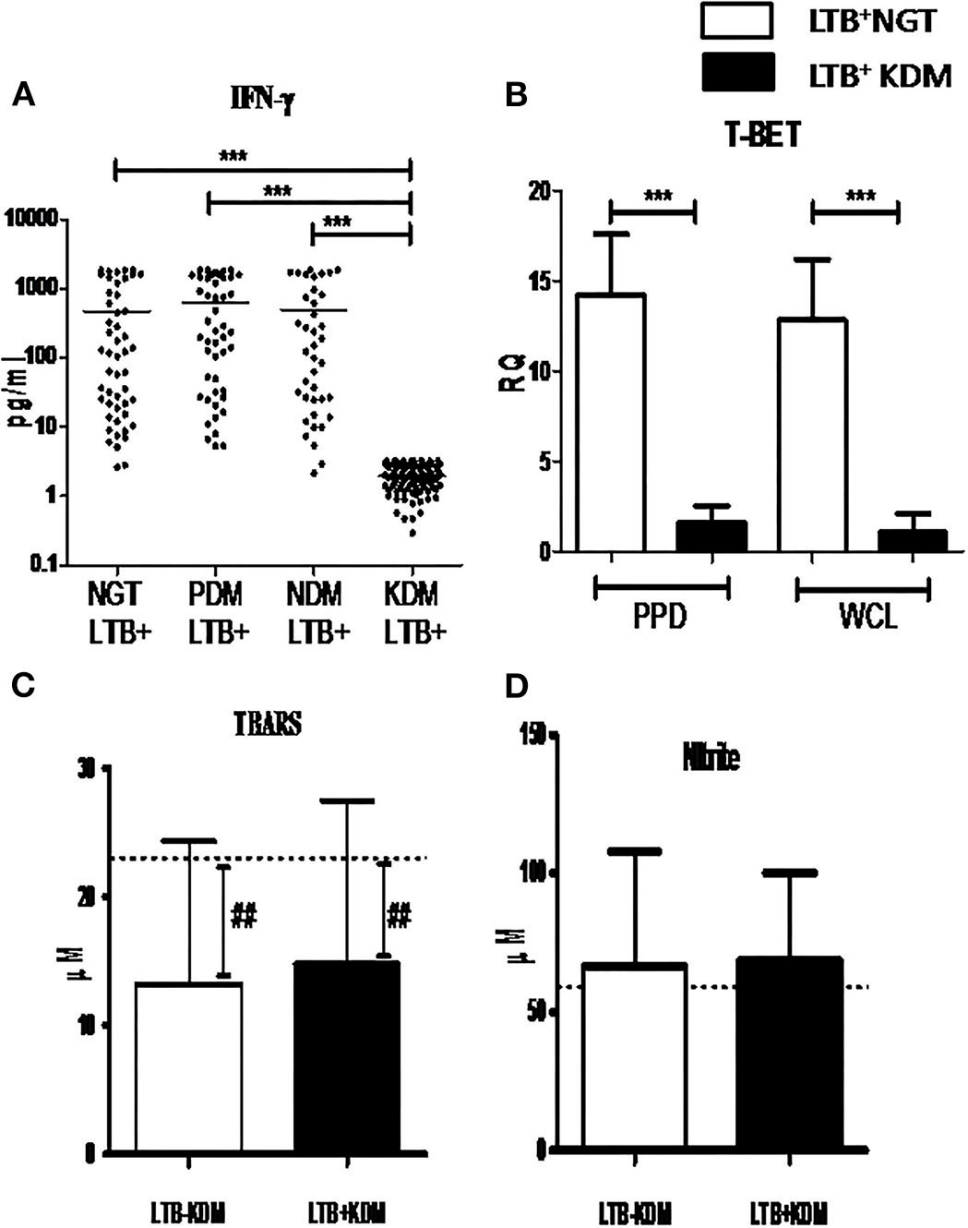
Chronic diabetes decreases the secretion of IFN-γ and expression of T-bet in LTB+ subjects. The levels of IFN-γ in the quantiferon supernatant in LTB+NGT (*n* = 50), LTB+PDM (*n* = 47), LTB+NDM (*n* = 39) and LTB+KDM (*n* = 76) subjects was quantified by ELISA **(A)**. The expression of T-bet in whole blood cultures stimulated with mycobacterial antigens (PPD and WCL) in LTB+NGT (*n* = 30) and LTB+KDM (*n* = 30) subjects w–as quantified by qRT-PCR **(B)**. The circulating levels of TBARS **(C)** and nitrite **(D)** in LTB-KDM (*n* = 93) and LTB+KDM (*n* = 82) subjects were quantified by colorimetry. The basal level, as was seen in LTB-NGT, is shown as dashed line (—-), as reference. Statistical significance was determined by non-parametric Mann–Whitney *U*-test and *p* < 0.05 was considered significant. *p < 0.05; **p < 0.01 for LTB-KDM vs. LTB+KDM; **##** *p* < 0.01 for LTB-NGT vs. LTB-KDM.

Next, in order to study the lack of increased prevalence of LTBI in the KDM group *M.tb* antigen-specific IFN-γ response was studied. IFN-γ secretion in the quantiferon culture supernatant (Figure 7A) and expression of T-Bet in whole blood cultures, after recall antigen challenge, was quantified (Figure 7B), in LTB^+^ subjects. No significant difference was seen in the basal level secretion between the study groups (data not shown). However, LTB^+^NGT, PDM, and NDM subject are secreted significantly higher levels of IFN-γ, following the recall antigen challenge, while LTB^+^KDM subjects failed to do so (Figure 7C). Furthermore, the antigen-induced expression of T-bet was significantly lower in LTB^+^KDM, compared to the LTB^+^NGT group (Figure 7B). No significant difference was seen in the TBARS (Figure 7D) and nitrite (Figure 7E) levels between LTB^+^NDM and LTB^−^NDM subjects. However, the levels were significantly reduced in both groups compared to healthy controls (LTB^−^NGT).

## Discussion

Diabetes patients with tuberculosis suffer from significant morbidity and mortality, which is called as “Diabetes–Tuberculosis Synergy” (Jeon and Murray, 2008; Sen et al., 2009). Hence, we expected a higher prevalence of LTBI among PDM, NDM, and KDM subjects. However, in contrast to our expectations, the study revealed several unexpected findings: (1) The prevalence of LTBI was significantly lower among PDM and NDM groups, compared to NGT and KDM; No difference was seen between NGT and KDM groups, (2) The decreased prevalence of LTBI, in the PDM and NDM groups, was associated with significant immunoendocrine modulation, and (3) In contrast to diabetes, a strong positive association was seen between LTBI and hypertension.

**Figure 8:**
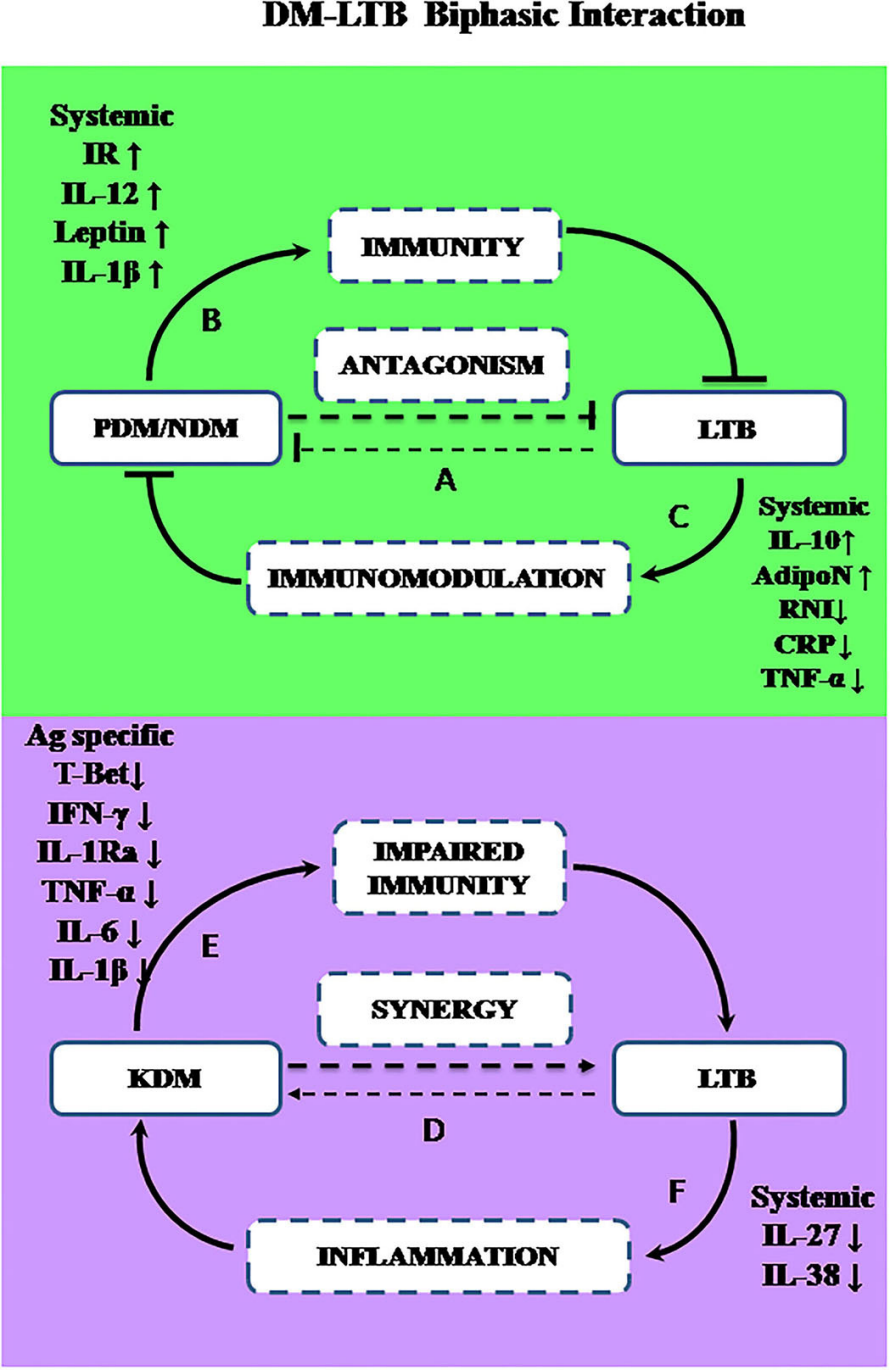
A biphasic model showing the complex interaction between diabetes and LTBI. The prevalence of LTBI was significantly decreased in PDM and NDM stages indicating antagonism **(A)**. During the early stages of diabetes (PDM and NDM), increased insulin resistance and increased circulating levels of IL-1β, IL-12, and leptin may promote immunity against LTBI **(B)**. Alternatively, LTBI through IL-10 mediated immunomodulation, can induce adiponectin secretion and reduce nitrite synthesis and can confer protection against diabetes **(C)**. However, chronic diabetes can promote LTBI infection or LTBI reactivation while LTBI can worsen glucose intolerance, indicating synergy **(D)**. In chronic diabetes (KDM), due to T-lymphocyte dysfunction, *M.tb* antigen-specific secretion of IFN-γ and T-bet expression are reduced, which can impair anti-TB immunity **(E)**. Alternatively, LTBI can promote inflammation by downregulation of anti-inflammatory cytokines like IL-27 and IL-38, leading to worsening of glucose tolerance **(F)**.

The most unexpected finding in the present study is the lower prevalence of LTBI among PDM and NDM subjects, compared to NGT and KDM groups. In a recent meta-analysis done on 13 studies (which includes both IGRA and TST based screening) only a very small risk factor was found to be associated with LTBI in diabetes (Lee et al., 2016). Being a cross-sectional study, at this juncture, it is difficult to explain this inverse relationship between diabetes and LTBI. Previously, we and others have reported such an inverse relationship between lymphatic filariasis and diabetes (Aravindhan et al., 2010; Wiria et al., 2012), which paved the way for the formulation of the “metabolic hygiene hypothesis” (Aravindhan and Anand, 2017). A common denominator between filariasis and LTBI is the immunomodulatory effect these infections can have on diabetes. Theoretically, LTBI can confer protection against pre-diabetes through immunomodulation leading to hormonal homeostasis; alternatively, pre-diabetes being a pro-inflammatory state, can confer protection against LTBI, due to high circulating levels of cytokines (Ruotsalainen et al., 2006; Wang et al., 2016). In order to explore this possibility, we carried out systemic cytokine and hormone profiling in the sub-groups of the study population. Among the various cytokines, pro-inflammatory cytokines (TNF-α, IL-6, and IL-1β), and Th1 cytokines (IL-12, IFN-γ, and IL-2) are known to confer significant protection against tuberculosis (Appelberg, 1994), while anti-inflammatory cytokines (IL-10 and TGF-β) (Etna et al., 2014) and type-I interferons (IFN-β) confer susceptibility (Moreira-Teixeira et al., 2020). Interestingly, most of these cytokines were also found to be elevated in pre-diabetes and were responsible for insulin resistance and pancreatic-beta cell destruction (Wei et al., 2008). As expected, serums levels of TNF-α, IL-6, IL-1β, and IFN-β were significantly elevated in PDM. Co-morbidity with LTBI specifically downregulated CRP and TNF-α and up-regulated IL-12 levels. Previously, we have reported increased levels of IL-10 in LTB^+^PDM subjects (Bobhate et al., 2021). The up-regulation of IL-10 and downregulation of TNF-α and CRP can augment insulin sensitivity, while increased levels of IL-12 can augment anti-TB immunity. Further, serum hormonal profiling indicates increased levels of adiponectin and leptin in LTB^+^PDM subjects, which can increase insulin sensitivity (Ahlstrom et al., 2017) and insulin release (Shimizu et al., 1997), respectively. Co-morbidity with LTBI also significantly reduced the nitrite levels in PDM subjects. It is important to note that “nitrosative stress” is an important contributor to the pathogenesis of diabetes (Al-Nimer et al., 2012). These findings indicate downregulation of inflammation, leading to increased insulin sensitivity, augmentation of anti-TB immunity, and hormonal homeostasis as major factors responsible for the inverse relationship between PDM and LTB (Figure 8).

Like PDM, NDM subjects also had a reduced prevalence of LTBI. However, the cytokine profile of PDM, NDM, and KDM did not follow the glycemic profile, indicating stage-specific immunomodulation, as we reported previously (Bobhate et al., 2021; Aravindhan et al., 2022). While serum TNF-α, IL-6, IL-1β, and IFN-β were significantly elevated in NDM, IFN-γ levels were significantly reduced. Co-morbidity with LTBI specifically up-regulated IL-1β, which is known to confer protection against TB (Mayer-Barber et al., 2014). The hormone profiling showed reduced levels of insulin, which was responsible for diabetes precipitation. Co-morbidity with LTBI, specifically upregulated the adiponectin levels, with no effect on insulin and leptin levels. Previously, we have reported increased levels of serum IL-10 in NDM-LTB^+^ compared to NDM-LTB^−^ (Bobhate et al., 2021). Overall, the cytokine and hormone profile again indicate downregulation of inflammation, augmentation of anti-TB immunity, and hormonal homeostatic, as major factors contributing to this inverse relationship, seen during the NDM stage (Figure 8).

Another unexpected finding in this study, is the same prevalence of LTBI in the NGT and KDM groups, while we expected an increase in the latter group, due to severe immune impairment (Masood et al., 2022). In order to understand the mechanism, we compared the IFN-γ levels in the quantiferon culture supernatants in LTB^+^ subjects, belonging to the four diabetic groups. There was no significant difference in the basal level secretion of IFN-γ between the groups (IFN-γ levels in TB antigen NIL tubes -data not shown). However, TB antigen-induced secretion of IFN-γ was drastically reduced in the LTB^+^ KDM subjects, indicating strong defects in adaptive immune responses. This was also accompanied by the lack of IL-1Ra (Bobhate et al., 2021) and IL-2 secretion (data not shown). The poor secretion of IFN-γ might account for the low detection rate of LTBI, among the KDM subjects. Thus, IGRA might increase the false negative cases among chronic diabetes subjects and might not be an ideal test for LTBI screening among these patients, as was reported previously (Shin et al., 2017). The poor secretion of IFN-γ was mainly due to the failure to upregulate T-bet expression, following antigenic stimulation. T-bet is a master regulator of Th1 differentiation and in the absence of which both Th1 differentiation and IFN-γ secretion are drastically altered (Sullivan et al., 2005). The defective adaptive immune response accounts for both the low detection rate of LTB and the possibility of high rates of endogenous reactivation, in this group, accounting for diabetes–tuberculosis synergy (Jeon and Murray, 2008; Sen et al., 2009). Furthermore, the KDM subjects had significantly reduced levels of TBARS (but not nitrites) as was reported previously (Chakraborty et al., 2011). The strong augmentation of adiponectin in LTB^+^ subjects in all the four groups is in accordance with a recent report (Ayyappan et al., 2018).

Finally, the most unexpected finding of this study was the strong association seen between LTBI and hypertension. In all the four groups, LTB^+^ subjects had higher SBP and DBP compared to LTB^−^ subjects. The prevalence of LTBI was much higher in the hypertensive group compared to the non-hypertensive group. Few studies have shown an association between extra pulmonary tuberculosis and pulmonary hypertension (Bhalla et al., 2010; Mojtahedzadeh et al., 2012). Recently, the importance of hypertension in active TB disease was reviewed with a limited number of studies (Seegert et al., 2017). Since the primary objective of our study was the diabetes status, a detailed history of hypertension (newly diagnosed vs. chronic, disease duration, treatment, etc) could not be collected. However, to the best of our knowledge, this is the first report to show a strong positive correlation between LTBI and hypertension and this obviously needs further exploration.

Recently, Mycobacterium has been shown to confer significant protection against heterologous diseases, by a mechanism called “trained immunity” (Gopalaswamy et al., 2020). In trained immunity, the innate immune cells, such as macrophages, DCs and NK cells, can be trained by mycobacterium to develop a pro-inflammatory phenotype, which can confer protection against infection by non-mycobacterial, fungal, or viral pathogens (Singh et al., 2020). However, the contribution of such a non-specific immune response, elicited by LTBI, in the setting of diabetes, is not fully understood. It is important to note that the immune status during active TB and LTB are quite different: while active TB is associated with a strong inflammatory reaction, LTB is associated with immunomodulation (Madan et al., 2021). Recently, in a small study carried out in India, LTBI was shown to confer significant protection against the severe form of the covid-19 disease (Madan et al., 2021). Similarly, the metabolic status of diabetes and pre-diabetes are quite different: while frank diabetes is associated with insulin resistance and deficiency, pre-diabetes is associated with insulin resistance and hyperinsulinemia. In contrast to diabetes and metabolic syndrome, isolated insulin resistance, under certain conditions, can confer some evolutionary advantage, at the organism level (Soeters and Soeters, 2012), and in this case anti-TB immunity. India has long been identified as an endemic zone for tuberculosis (Pathak et al., 2021) and is competing with China in becoming the diabetes capital of the world (Joshi and Parikh, 2007). The active implementation of the national end TB (NETB) program in India over the past several years might have reduced the LTBI burden (Dolla et al., 2019; Bhargava et al., 2021), which could have gradually contributed to the increase in diabetes, over the years (“metabolic hygiene hypothesis”) (Aravindhan and Anand, 2017). Further studies are thus needed to substantiate this inverse relationship between diabetes and LTBI.

## Conclusion

The most interesting finding in this study is that the relationship between diabetes-latent tuberculosis is complex and biphasic: At the early stages of diabetes, it is reciprocal; while at a late stage it is synergistic—this important phenomenon obviously needs further research (Figure 8). Cytokine and hormone profiling indicated that IL-10 mediated secretion of adiponectin could be the most important mechanism by which LTBI can confer protection against insulin resistance. Insulin resistance which was associated with high circulating levels of IL-12, leptin, and IL-1β could be the most possible reason behind anti-TB immunity. During chronic diabetes, a synergistic interaction was seen with LTBI. The reduced expression of T-Bet and defective antigen-specific IFN-γ response, as seen in the KDM group, could account for both the low detection rate of LTBI and the high probability of endogenous reactivation. Thus, the interaction between diabetes and LTBI is complex involving a multifaceted immune-endocrine mechanism.

## Authors Summary

Diabetes patients have impaired immunity which makes them more susceptible to tuberculosis. In general, diabetes patients have higher sputum bacillary load, delayed sputum clearance, high rates of treatment failure, higher incidence of multi-drug resistance, increased cavitary lung lesion, etc. This is called as diabetes-tuberculosis nexus, which could either be due to an increased infection rate or increased reactivation of latent tuberculosis infection (LTBI). In the present study, we looked at the prevalence of LTBI among subjects with various stages of glucose intolerance. In contrast to our hypothesis, the prevalence of LTBI was the same between control subjects and chronic diabetes patients, while it was significantly reduced in pre-diabetes and newly diagnosed diabetes patients. Further, hypertension which is a common co-morbidity seen with diabetes was found to be a major risk factor for LTBI, which hasn't been reported to date. Apart from prevalence, we also studied the disease mechanism by quantifying circulating levels of cytokines, hormones, and redox markers. Our study indicated modulation of the immunoendocrine system, as a probable mechanism behind the biphasic relationship between LTBI and diabetes.

## Data Availability Statement

The raw data supporting the conclusions of this article will be made available by the authors, without undue reservation.

## Ethics Statement

The studies involving human participants were reviewed and approved by MV Hospital Institutional Human Ethical Committee. The patients/participants provided their written informed consent to participate in this study.

## Author Contributions

VA and VV conceived and designed the experiment, drafted the manuscript, and garantors of the data. AB and KS performed the experiment. VA, SK, and AP analyzed the data. VA, AB, AP, and VV contributed to the discussion and reviewed the manuscript. All authors contributed to the article and approved the submitted version.

## Funding

This work was supported by Department of Biotechnology, New Delhi (BT/PR5693/MED/29/585/2012). The funders had no role in study design, data collection and analysis, decision to publish, or preparation of the manuscript.

## Conflict of Interest

The authors declare that the research was conducted in the absence of any commercial or financial relationships that could be construed as a potential conflict of interest.

## Publisher's Note

All claims expressed in this article are solely those of the authors and do not necessarily represent those of their affiliated organizations, or those of the publisher, the editors and the reviewers. Any product that may be evaluated in this article, or claim that may be made by its manufacturer, is not guaranteed or endorsed by the publisher.
